# Lower Limb Maneuver Investigation of Chasse Steps Among Male Elite Table Tennis Players

**DOI:** 10.3390/medicina55040097

**Published:** 2019-04-08

**Authors:** Changxiao Yu, Shirui Shao, Jan Awrejcewicz, Julien S. Baker, Yaodong Gu

**Affiliations:** 1 Department of Human Movement Science, Faculty of Sports Science, Ningbo University, Ningbo 315211, China; lizhongzheng9@foxmail.com (C.Y.); shaoshirui@nbu.edu.cn (S.S.); 2 Department of Automation, Biomechanics and Mechatronics, Lodz University of Technology, 90-924 Lodz, Poland; jan.awrejcewicz@p.lodz.pl; 3 School of Health and Life Sciences, University of the West of Scotland, Hamilton ML3 OJB, UK; jsbaker@uws.ac.uk

**Keywords:** table tennis, chasse step, kinematics, EMG, biomechanics

## Abstract

*Background and Objectives:* The popularity of table tennis has increased globally. As a result, the biomechanical movement patterns in the lower limb during table tennis have attracted extensive attention from coaches, scientists and athletes. The purpose of this study was to compare the differences between the long and short chasse steps in table tennis and evaluate risk factors related to injuries in the lower limb. *Materials and Methods:* Twelve male elite athletes performed forehand topspin strokes with long and short chasse steps in this study, respectively. The kinematics data of the lower-limb joints were measured by a Vicon motion analysis system. The electromyograms (EMG) of six lower-limb muscles were recorded using a myoelectricity system. *Results:* The key findings were that the angle change rate of the ankle in the long chasse step was faster with a larger range of motion (ROM) in the coronal and transverse planes. The hip was also faster in the sagittal and transverse planes but slower in the coronal plane compared with the short chasse step. In addition, the vastus medialis (VM) was the first activated muscle in the chasse step. *Conclusions:* The hip and ankle joints in the long chasse step and the knee joint in the short chasse step have higher susceptibility to injury. Moreover, tibialis anterior (TA), vastus medialis (VM) and gastrocnemius (GM) should be sufficiently stretched and warmed prior to playing table tennis. The results of this study may provide helpful guidance for teaching strategies and providing an understanding of potential sport injury mechanisms.

## 1. Introduction

As a sport, table tennis has become one of the most popular sports with over 300 million supporters worldwide [1]. Table tennis is not only a complex and asymmetric sport, but has various tactics that utilize speed and direction changes. Agile footwork is a rational movement that enables an athlete to rapidly change his/her position/direction and regulate his/her body for a particular stroke in order to achieve maximal power and effect [2]. Essentially, footwork is the foundation of table tennis techniques [3]. Through a comparison of the shot characteristics between Asian and European top-level table tennis players, Malagoli Lanzoni et al. [4] found that the performance of strokes and footwork in Asians was quicker, more efficient and more frequent compared with Europeans. According to a previous report, the chasse step is considered to be one of the most frequently used attacking footwork movement patterns with a frequency of 15.2% in competitions [5]. The frequency of utilization of the chasse steps likely increases with a more specialized development of table tennis. Based on the positions of the balls landing on the table, athletes should perform different movements according to the distance (long and short chasse step). As a source of energy, the lower limb can transfer the optimum activation from the lower body segments to the upper limb through sequential movements of the kinetic chain [6,7]. Previous studies have documented that a complex stroke motion requires stability and flexibility in the lower limb, which could effectively increase the racket speed [8]. In terms of the characteristics of performances in table tennis, athletes have to complete a series of complex spatial movements that include acceleration, deceleration, direction change, moving quickly and balance control. All of these varied and different movement patterns help athletes to generate their optimum stroke production [9]. Therefore, in order to perform the chasse step efficiently in table tennis, athletes and coaches should pay more attention to its characteristics and mechanisms. The kinematics of the chasse step patterns in athletes’ lower limbs are more interesting and it is important to determine whether this motion increases the risk of sport injuries. How the activation sequence of the muscles in the body segment provides movement is also of great interest.

During a complex motion, body segments must be coordinated in a perfect sequence that is defined as the “kinematic chain” [10,11,12]. This may contribute to generating more power and better control when returning the balls with chasse steps in certain positions, which is based on the different landing positions of the ball on the table. After researching the angular kinematics between the cross-court and long-line shots during forehead top spin moves for table tennis, Malagoli Lanzoni et al. [13] reported that the powerful torsion of the trunk would contribute to an increase in the development of speed during the racquet swing, which means that the maximum speed of the racket would occur at the ball/racket impact moment. Iino and Kojima [14] showed that the racket speed was not only dictated by skill level but more importantly by the lower trunk axial rotation movement in table tennis. Therefore, as the origin of the kinematic chain, the drive in the lower limb can significantly affect the swing quality in racket sports [7,12,15]. Iino and Kojima [16] stressed that the hip motion played a key role in trunk rotation while Qian et al. [8] reported that the hip joint was vital in power generation and transmission in the lower limb drive. Seeley et al. [15] stated that the hip and ankle were important factors in the kinematic chain. Moreover, the characteristics of the kinematic chain revealed that the maximum velocities gradually transfer from the knee to the racquet [12,17,18]. These studies were consistent in the view that achieving good table tennis technique requires an optimum activation of all the links in the kinematic chain.

There are risk factors related to injury in all sports. Previous studies showed that the injury rate of the lower limb was higher than the upper extremity, which ranges from 39–59% of the total presented injuries (lower limb, upper extremity and the central core injuries) [19,20]. Kibler [21,22] found that any disruption of the kinematic chain could induce an increased loading of other joints in the sequential movements of body segments. Lam et al. [23] investigated the footwork effects on lower-limb kinematics and kinetics, which would provide useful guidance for the creation of training protocols and further understanding of the injury mechanisms. Generally, stretching is widely considered to be an effective warm-up strategy before strenuous exercise [24,25]. Previous studies showed that electromyography (EMG) can be used to detect the patterns of muscle activation in the upper and lower limb in racket sports [18,26,27,28]. Therefore, EMG measurement may be used to identify which muscles play primary roles in the chasse step movement patterns when playing table tennis. This knowledge can be used to improve performance characteristics and reduce the possibility of muscle strains. This may subsequently inform future prevention strategies, especially the pre-exercise warm-up. According to previous studies, females might have different psychological attitudes and different perceptions compared to their male counterparts [29]. This could influence performance outcomes. However, this study only focused on the chasse step based on the position of the balls landing on the table.

The aim of the study was to investigate the biomechanics between long and short chasse steps in table tennis and evaluate any potential injury risk factors for the lower limb. It was hypothesized that a short chasse step would require a shorter amount of time compared to the long chasse step during the chasse step movement. The long chasse step would show larger ROM and faster angle change rate compared with the short chasse step. It was also hypothesized that vastus medialis might be the first activated muscle during the chasse step.

## 2. Methods

### 2.1. Participants

Twelve elite male national level players (age: 20.64 ± 1.42 years; height: 1.74 ± 0.03 m; body mass: 67.73 ± 3.31 kg and training experience: 12.7 ± 1.5 years) participated in this study. All participants did not have any previous lower limb injuries and surgeries or foot diseases. This study was approved by the Ethics Committee of Ningbo University (No. 2016FS021) and the participants were informed about the experimental procedures and requirements. Written consent was obtained before the experimental data collection.

In order to determine the dominant lower limb, the ball-kick test was used in this study [30]. In this test, each participant was asked to kick a football with arbitrary power and maximal accuracy through a set of obstacles placed 1 m apart and 10 m from the participants. The supporting leg was regarded as a non-dominant limb and the other side was the dominant limb when kicking the football. In addition, this study followed the suggestions of Peters and Murphy [31] to determine the handedness and selected the right-handed athletes as the participants.

### 2.2. Experimental Setup and Data Processing

**Figure 1:**
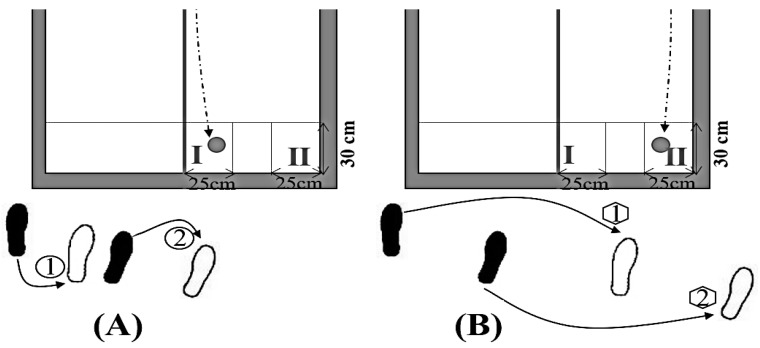
Short and long chasse steps and table designs. I and II are the different landing positions of the balls, respectively. A and B mean short and long chasse steps, respectively. ①, ② and 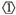, 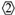 represent the footwork orders for chasse steps.

Testing sessions were conducted in the Ningbo University table tennis training center. A table tennis machine (Double Fish Sports Goods Group Co., Guangzhou, China) was placed 1.2 m away from the opponent’s court and presented topspin balls to the participants. The velocity, projecting angle and the frequency were consistent for all serving balls (Butterfly, Tokyo, Japan). Before testing, the participants were given a standardized warm-up of 20 min within the experimental environment. All participants were informed of the test purposes and procedures and used their own racquets (backplane:butterfly; forehand rubber:hurricane 3; backhand:tenergy 05). During testing, the participant first performed forehand returns to the service 2–4 times at the left of table before hitting the A or B region balls (Figure 1) using a forehand loop with the chasse step recorded by a Vicon motion analysis system (Oxford Metrics Ltd., Oxford, UK), separately. Participants hit the balls at each region (A or B) until 20 acceptable motions were accomplished. An eight-camera Vicon motion analysis system (Oxford Metrics Ltd., Oxford, UK) was used to capture the lower-limb kinematic data with a frequency of 200 Hz, while sixteen standard markers were adhered at the anterior–superior part of the iliac spine, posterior–superior part of the iliac spine, lateral mid-thigh, lateral knee, lateral mid-shank, lateral malleolus, second metatarsal head and calcaneus of the lower limb. An 8-channel surface EMG (ME6000, Mega Electronics, Kuopio, Finland) was used to collect the surface EMG signals from biceps femoris (BF), gluteus maximus (GM), rectus femoris (RF), gastrocnemius (GN), vastus medialis (VM) and tibialis anterior (TA) at a sampling frequency of 1000 Hz. The surface of the skin was prepared by cleaning and shaving, using an isopropyl alcoholic swab before wearing the myoelectric pads. An electrical muscle stimulator was used to locate each muscle’s belly. The different equipment did not affect the hitting actions of the players.

### 2.3. Date Recording and Statistics Analysis

The whole motion phase was collected and two key phases were identified for the subsequent joint angle analysis. The chasse step referred to two certain events, which include the takeoff instant (TI) of the dominant foot on the initial position and the backward-end (BE) moment on the completed position. The sagittal, coronal and horizontal ROM and angle change rate in the lower limb joints were recorded for each participant during one entire cycle. The joint angles in three planes were time-normalized to 100 data points for subsequent analysis.

In order to obtain the maximum EMG levels, each participant performed 10 chasse steps with comparable efforts. First, EMGs were band-pass filtered (high-pass 20 Hz, low-pass 450 Hz) and full-wave rectified before the EMG signals using a root-mean-square (RMS) amplitude with a 40 s moving window were used to represent muscle activation. For each muscle, the onset of muscle activation was defined as an increase in the EMG signal amplitude that is greater than maximum EMG levels of 25% during one chasse step, lasting longer than 50 ms [32]. SPSS version 17.0 software (SPSS Inc., Chicago, IL, USA) was used for statistical analysis. An initial Shapiro–Wilk test confirmed that the data were normally distributed. To examine the differences between the two movements, a paired *t*-test was performed for each variable, including the joint angles at TI and BE moments and the time of the entire motion and joint ROM. One-way ANOVA was used to analyze the differences of angle change rate at three planes during the long stroke. The significance level was set at *p* < 0.05. According to the previously described equations, Cohen’s d was used to compare the differences in the average of the two groups and is often represented by the effect size [33]. The effect size (ES) is evaluated as being trivial (≤0.19), small (≥0.2 and ≤0.49), medium (≥0.50 and ≤0.79) and large (≥0.80), respectively [33]. The standard error of measurement (SEM) was used to be standard error of the mean. The statistical method to calculate RMS amplitude is provided as follows (see [34]):

(1) $$\sqrt{\frac{1}{N}\sum_{i-1}^{N}EMG{\left(i\right)}^{2}}$$

where *N* represents the data length and *i* represents the amplitude.

## 3. Results

**Figure 2:**
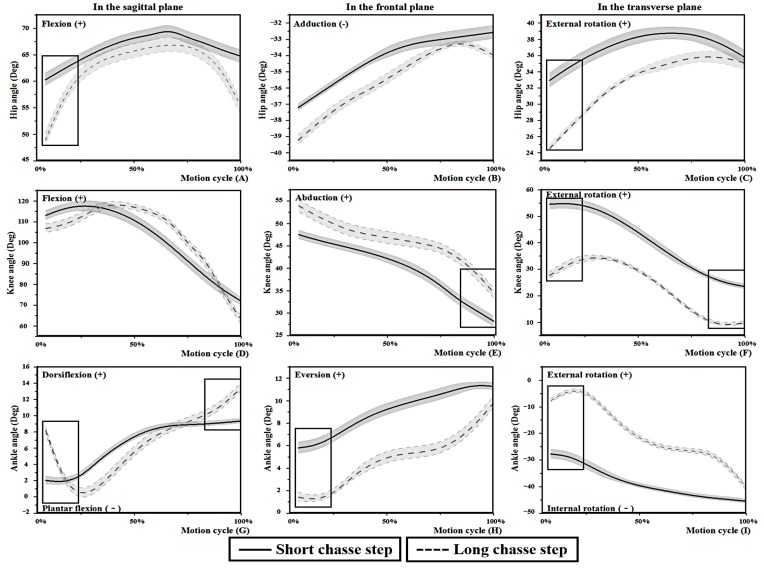
The differences in hip (**A**–**C**), knee (**D**–**F**) and ankle (**G**–**I**) angles during one motion cycle in three planes. “▯” means *p* < 0.05 between the two chasse steps at key events.

The time for performing the two chasse steps were 0.52 ± 0.02 s and 0.59 ± 0.01 s (*p* < 0.001) for the short and long strokes, respectively. The changing trend of the kinematics of the three-dimensional space was shown in the Figure 2 between the short and long chasse steps with generally comparable. As Table 1 shows, the ROM of the hip joint for the long chasse step was larger in the sagittal and transverse planes. The ROM of the ankle also showed larger differences in the coronal and transverse planes but the ROM of knee was smaller in the coronal plane compared with the short chasse step. In Figure 2, the peak joint angles are shown and they mostly occurred at the TI or the BE.

Significant differences in the key events were found in all three planes between the long and short chasse steps, particularly at the TI moment (Table 2). Compared to the short chasse step, the long chasse step required smaller hip flexion and external rotation; larger ankle dorsiflexion but smaller eversion and internal rotation; and smaller knee external rotation at the TI moment. Moreover, there were differences for the BE moment in the knee and ankle joints. The long chasse step showed larger knee abduction and ankle dorsiflexion but smaller knee external rotation than the short chasse step.

**Figure 3:**
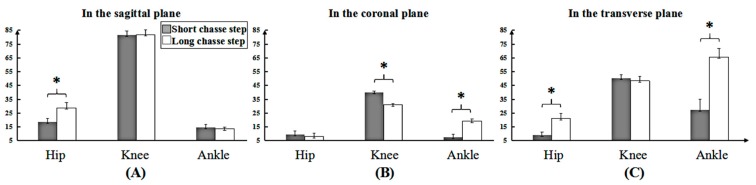
The angle change rate (Deg/s) between long and short chasse steps in three planes. (**A**–**C**) shows the sagittal, coronal and transverse plane, respectively. * shows the differences in the hip, knee and ankle joint, respectively.

As shown in Figure 3, the angle change rate for the long chasse step in the hip joint was faster than that for the short chasse step in the sagittal and transverse planes. However, a slower angle change rate of the knee joint for the long chasse step existed in the coronal plane compared to that in the short chasse step. The angle change rate at the ankle joint for the long step was faster than that for the short chasse step in the coronal and transverse planes.

**Figure 4:**
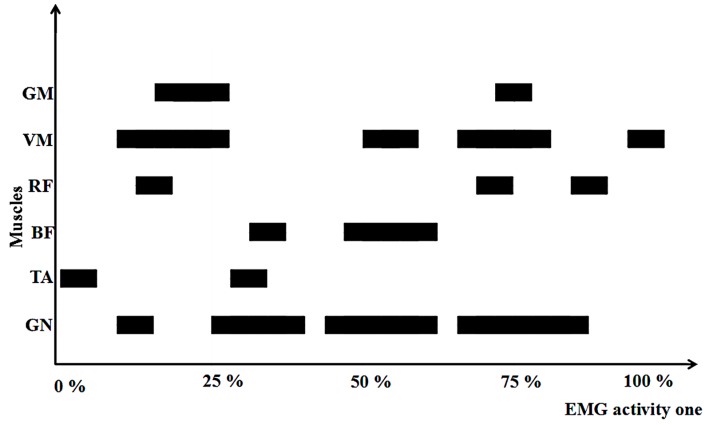
The trend of the activation sequence of the six muscles during one chasse step.

Figure 4 shows the differences in the activation sequence of the six muscles across an entire chasse step. Differences were observed in muscle timing. TA was activated first, followed by VM and GN. Finally, BF was the last to be activated. This would be useful for coaches and athletes when they consider warm-up strategies.

## 4. Discussion

This study compared the biomechanics of the lower limb between the long and short chasse steps in table tennis and observed the activation sequence of the selected six muscles of the lower limb. Two key technique events (takeoff instant, TI and backward-end, BE) and an entire chasse step was identified for the indepth analysis. The results showed that the short chasse step showed a faster completion time compared to the long chasse step. Significant differences in kinematics were found in terms of a larger ROM and faster angular changing rate for the long chasse step compared with the short chasse step. There were significant differences in the hip and ankle joints between the two chasse steps at the TI moment, but both of them at the BE moment showed significant differences at the knee joint. The VM muscle was the first activated muscle during one entire chasse step. The hypothesis is supported by the results that there are significant differences in kinematics and EMGs between the long and short chasse steps.

For the short chasse step in the TI of the dominant foot, the hip movement exhibited larger flexion and external rotation compared to the long chasse step. Qian et al. [8] emphasized that the movement of hip joint is a vital factor for energy generation and energy transference in the kinetic chain during table tennis. Due to the shorter time compared with the long chasse step, the lower center of gravity for the short chasse step would contribute to an efficient switching to the next phase. The short chasse step also showed a larger knee external rotation, ankle eversion and external rotation. Previous studies demonstrated that the peak joint angles of hip flexion and larger knee external rotation during backswing could improve the ball speed in racket sports [15,35]. Moreover, the larger ankle eversion and external rotation would provide a more stable base during the short chasse step. According to a previous study, peak pressures were higher under the medial forefoot of the dominant foot during the chasse step [3]. Thus, this might cooperate with larger ankle dorsiflexion of the long chasse step to potentially increase the ground reaction force that would improve technical performance. Based on the above analysis, the short chasse step exhibited a better foundation than the long chasse step, which could lead to a more flexible and fuller backswing.

In the BE moment, the long chasse step showed larger knee abduction and ankle dorsiflexion but smaller knee external rotation compared with the short chasse step. Based on the theory of the stretch-shortening cycle, which states that prior stored elastic energy in the muscle-tendon stretching phase could increase concentric movement [7,8,36,37], Komi and Bosco [36] reported the presence of significant elastic energy storage for a squatting jump compared with a drop jump and counter-movement jump at the starting position. This is consistent with the present findings as greater knee external rotation at the BE moment may contribute to and facilitate the stretching of the internal rotator, which would improve the contraction effect for the forward swing. Furthermore, Elliott et al. [38] explained that players with better lower limb inertia may transfer this to the upper limb to achieve maximal external rotation. Larger knee abduction and ankle dorsiflexion were also found at the BE moment, which is an important factor for table tennis players to control their body posture and maintain balance.

The complex structure and function of the lower-limb joints are essential for effective footwork in table tennis players. Sports injuries often occur during sport activities (training and competition) and the role of physicians goes beyond mere diagnosis and injury treatment. Physicians are required to take part in the rehab process by providing appropriate training advice and they help athletes to quickly return to practicing sport and help to decrease the risk of potential injuries. An indepth understanding of the kinematics during the long and short chasse steps could improve the effectiveness of training. The results of the present study demonstrate that the long chasse step showed larger ROM compared with the short chasse step and thus, we can infer that the long chasse step required more flexible movements of the lower limb joints. However, the greater ankle internal rotation could induce the potential risk of ankle sprain [8,39] and Achilles tendinitis [40]. Additionally, the larger ROM of ankle joint eversion also increased the risk of sport ankle sprains as reported by Beynnon et al. [41]. The greater hip ROM can contribute to a shift in the weight, which can also facilitate momentum generation [42]. Moreover, there is an optimal combination of the surrounding muscle, ligament, articular surfaces, tendon and joint capsule laxity [43], which promotes a stable center of mass shift in the long chasse step. For joint angular velocity generation, it is also necessary to understand the importance of optimizing energy transfer in the kinetic chain [15]. However, fast pivoting and landing induces lower limb injuries more easily [44]. Compared with the short chasse step in the sagittal and transverse planes, the change rate of the angle at the hip joint for long chasse steps was clearly faster, while the change rate of the angle at the knee joint in the frontal plane was slower. In the transverse plane, the change rate of angle at ankle joint was faster for the long chasse step than the short chasse step. However, lateral ankle sprains occurred at high twisting forces [20,45]. Moreover, the knee is susceptible to overuse and overload injuries [20]. Based on the analysis of the results, the hip and ankle joints of the long chasse step and the knee joint of the short chasse step may result in a higher susceptibility to injury.

To our knowledge, the present study is the first to have reported lower limb EMG and kinematics in table tennis players across the entire chasse step. The report of Kondrič et al. [46] found that the most frequent injuries in racket sports occurs in muscle tissues during training and/or competition processes, with a high percentage of injuries found in lower-limb joints (ankle and foot in particular; 23.69% in total). They also indicated that due to the characteristics of abrupt blocking movements in playing table tennis, there is a high percentage of hip injuries (5.76%). Strains and sprains were the most common type of injury in the majority of sports [19]. The results showed that TA was the first muscle activated during chasse step, followed by the VM and GN muscles. In the thigh areas, the adductor muscles and the hamstrings have a high risk of strain injuries, particularly during sudden direction changes [20]. Moreover, the knee ROM in the coronal plane was higher in the short chasse step than the long one. This increased knee hyperextension might increase the risk of the anterior cruciate ligament injury [47,48]. Gastrocnemius muscle strains commonly occurred during repetitive and explosive accelerations of the lower limb, particularly when using sprinting or jumping movements [20]. In order to reduce the incidence of muscular strains and sprains, this study suggests that creating some warm-up exercises for TA, VM and GN muscles may be beneficial.

However, there are some limitations that should be mentioned in this study. First of all, a small sample size may limit the external validity to some degree. Secondly, this study did not compare the relationship between the skill level and gender between the long and short chasse steps. Finally, caution is suggested in terms of the generalization and application of these findings to players from other countries. This is related to the fact that the participants in this study were all from China.

## 5. Conclusions

For the short chasse step, the knee may be at a higher risk of sport injury in the frontal plane. For the long chasse step, the hip in the sagittal and transverse planes and the ankle in the transverse plane could increase the risk of sport injuries. Therefore, some pertinent protective measures should be adopted when table tennis players practice the long or short chasse steps. These may include the systematic strengthening of the muscle involved in the different chasse steps. Coaches and players also should pay more attention to flexibility training for tibialis anterior, vastus medialis and gastrocnemius muscles during the pre-exercise warm-up.

## Figures and Tables

**Table 1:** Mean ± standard deviations (mean ± SD), standard error of measurement (SEM), 95% confidence intervals (CI), effect sizes (ES) for the lower-limb joints ROM of the motion between long and short chasse steps.

Short or Long Chasse Steps		Mean ± SD	SEM	CI	ES
Hip					
X	Short	9.57 ± 1.30	0.38	(8.74, 10.40)	0.83
	Long	15.11 ± 2.34 *	0.68	(13.62, 16.60)	
Y	Short	4.91± 1.26	0.36	(4.11, 5.71)	0.31
	Long	4.12 ± 1.17	0.34	(3.38, 4.86)	
Z	Short	4.70 ± 1.06	0.31	(4.03, 5.38)	0.91
	Long	11.08 ± 1.82 *	0.52	(9.92, 12.23)	
Knee					
X	Short	42.23 ± 1.60	0.46	(41.21, 43.24)	0.20
	Long	42.87 ± 1.60	0.46	(41.85, 43.88)	
Y	Short	20.70 ± 1.67	0.48	(19.64, 21.77)	0.83
	Long	16.22 ± 1.27 *	0.37	(15.41, 17.03)	
Z	Short	26.00 ± 1.37	0.40	(25.13, 26.87)	0.22
	Long	25.39 ± 1.39	0.40	(24.51, 26.27)	
Ankle					
X	Short	7.66 ± 1.11	0.32	(6.95, 8.36)	0.06
	Long	7.10 ± 0.71	0.21	(6.64, 7.55)	
Y	Short	3.82 ± 1.16	0.34	(3.08, 4.56)	0.95
	Long	10.00 ± 0.80 *	0.23	(9.49, 10.51)	
Z	Short	14.11 ± 3.83	1.12	(11.68, 16.55)	0.94
	Long	34.37 ± 3.22 *	0.93	(32.32, 36.42)	

X = the sagittal plane; Y = the coronal plane; and Z = the transverse plane. * *p* < 0.05 in the hip, knee and ankle.

**Table 2:** Mean ± standard deviations (Mean ± SD), standard error of measurement (SEM), 95% confidence intervals (CI), effect sizes (ES) for the comparison of joint angles in two key moments in three planes between short and long chasse steps.

			Short Chasse Step			Long Chasse Step			ES
			Mean ± SD	SEM	CI	Mean ± SD	SEM	CI	
TI	Hip	X	60.16 ± 2.42	0.70	(58.62, 61.70)	48.85 ± 3.22 *	0.93	(46.80, 50.89)	0.89
		Y	−37.78 ± 0.94	0.27	(−38.38, −37.18)	−38.30 ± 1.39	0.40	(−39.18, −37.41)	0.21
		Z	33.01 ± 1.57	0.45	(32.02, 34.01)	24.53 ± 0.36 *	0.10	(24.30, 24.76)	0.97
	Knee	X	109.79 ± 3.38	0.97	(107.65, 111.94)	107.95 ± 1.42	0.41	(107.05, 108.86)	0.33
		Y	49.96 ± 2.00	0.58	(48.69, 51.23)	51.08 ± 2.04	0.59	(49.78, 52.38)	0.27
		Z	54.15 ± 1.75	0.51	(53.04, 55.27)	29.26 ± 1.77 *	0.51	(28.13, 30.39)	0.99
	Ankle	X	2.06 ± 1.31	0.38	(1.23, 2.89)	8.21 ± 1.07 *	0.31	(7.53, 8.89)	0.93
		Y	5.81 ± 1.02	0.29	(5.16, 6.46)	1.48 ± 0.75 *	0.22	(1.00, 1.96)	0.92
		Z	−27.10 ± 2.43	0.70	(−28.64, −25.56)	−11.72 ± 1.90 *	0.55	(−12.92, −10.51)	0.96
BE	Hip	X	64.45 ± 2.82	0.81	(62.66, 66.24)	55.14 ± 3.26 *	0.94	(53.07, 57.20)	0.84
		Y	−32.86 ± 1.49	0.43	(−33.81, −31.92)	−33.72 ± 2.03	0.59	(−35.01, −32.43)	0.23
		Z	35.33 ± 1.59	0.46	(34.32, 36.34)	35.60 ± 2.02	0.58	(34.32, 36.88)	0.07
	Knee	X	69.24 ± 1.85	0.53	(68.06, 70.41)	69.08 ± 2.13	0.61	(67.73, 70.44)	0.04
		Y	29.25 ± 1.88	0.54	(28.06, 30.45)	34.86 ± 1.45 *	0.42	(33.94, 35.78)	0.86
		Z	28.77 ± 1.95	0.56	(27.54, 30.00)	9.86 ± 0.65 *	0.19	(9.45, 10.27)	0.99
	Ankle	X	9.72 ± 0.68	0.20	(9.29, 10.15)	10.39 ± 0.54 *	0.15	(10.05, 10.73)	0.48
		Y	9.63 ± 0.90	0.26	(9.06, 10.20)	9.99 ± 0.88	0.26	(9.42, 10.55)	0.20
		Z	−41.21 ± 2.42	0.70	(−42.75, −39.67)	−40.59 ± 3.23	0.93	(−42.64, −38.54)	0.11

TI = takeoff instant of dominant foot; BE = backward-end. X = the sagittal plane; Y = the coronal plane; and Z = the transverse plane. * *p* < 0.05 in the hip, knee and ankle.
